# Revealing the Effect of Heat Treatment on the Spectral Pattern of Unifloral Honeys Using Aquaphotomics

**DOI:** 10.3390/molecules27030780

**Published:** 2022-01-25

**Authors:** Zsanett Bodor, Csilla Benedek, Balkis Aouadi, Viktoria Zsom-Muha, Zoltan Kovacs

**Affiliations:** 1 Department of Measurements and Process Control, Institute of Food Science and Technology, Hungarian University of Agriculture and Life Sciences, 14-16 Somlói Street, H-1118 Budapest, Hungary; bodor.zsanett@phd.uni-mate.hu (Z.B.); aouadi.balkis@phd.uni-mate.hu (B.A.); zsomne.muha.viktoria@uni-mate.hu (V.Z.-M.); 2 Department of Dietetics and Nutrition, Faculty of Health Sciences, Semmelweis University, 17 Vas Street, H-1088 Budapest, Hungary; benedek.csilla@se-etk.hu

**Keywords:** honey, heat treatment, NIRS, chemometrics, aquagram, PCA, PCA-LDA, PLRS, WAMACs, HMF

## Abstract

In this study we aimed to investigate the effect of heat treatment on the spectral pattern of honey using near infrared spectroscopy (NIRS). For the research, sunflower, bastard indigo, and acacia honeys were collected from entrusted beekeepers. The honeys were not subject to any treatment before. Samples were treated at 40 °C, 60 °C, 80 °C, and 100 °C for 60, 120, 180, and 240 min. This resulted in 17 levels, including the untreated control samples. The 5-hydroxymethylfurfural (HMF) content of the honeys was determined using the Winkler method. NIRS spectra were recorded using a handheld instrument. Data analysis was performed using ANOVA for the HMF content and multivariate analysis for the NIRS data. For the latter, PCA, PCA-LDA, and PLSR models were built (using the 1300–1600 nm spectral range) and the wavelengths presenting the greatest change induced by the perturbations of temperature and time intervals were collected systematically, based on the difference spectra and the weights of the models. The most contributing wavelengths were used to visualize the spectral pattern changes on the aquagrams in the specific water matrix coordinates. Our results showed that the heat treatment highly contributed to the formation of free or less bonded water, however, the changes in the spectral pattern highly depended on the crystallization phase and the honey type.

## 1. Introduction

Honey, as a natural sweetener used since ancient times [1], is a perfect candidate for the increasing demand for unprocessed, natural, and “healthy” products. According to the literature, honey is produced by honeybees (*Apis mellifera*) from the nectar and sap of living plants or honeydew [2,3]. Owing to its valuable nutritional composition and relatively high price on the market, it represents an important value for the consumers. Honey is composed of sugars (95% of the dry matter content [4]), water (<20%) and numerous nutritious compounds, such as minerals, amino acids, vitamins, and phytochemicals [5,6]. The composition of honey is highly influenced by both botanical and geographical origin [6]. However, there are other factors—for example storage conditions or processing—that can have an impact on the composition and sensory characteristics (e.g., aroma, color) of honey [7,8]. The processing of honey includes heat treatment, which is often applied for the elimination of the crystals or to delay crystallization.

Honey crystallization is a naturally occurring process, depending mainly on the ratio of fructose and glucose and the water/glucose fraction. Other factors, such as the presence of pollen and other particles can also influence the crystallization process. Honeys with a higher glucose content (28–30 g/100 g), fructose/glucose ratio < 1.14 and/or glucose/moisture ratio ≥ 2.1 crystallize faster. The crystals are usually not preferred by the consumers due to the unpleasant organoleptic properties of the (rough) crystals [9,10]. Moreover, the crystallized state makes the handling of honey more difficult for the producers and beekeepers. Therefore, heat treatment can be applied for the liquefaction of honey, for which numerous methods are available [11]. The most common techniques are the treatment in chambers by hot air or water bath. Nowadays, different types of honey heater equipments are also available [12,13]. According to Hungarian regulations, the core temperature of honey cannot exceed 40 °C during the processing of honey [14], because at higher temperature levels the composition of honey changes significantly [15]. Other adverse aspects are also noticeable, including, but not limited to, changes of taste/aroma [16,17,18], decay of vitamins [19], changes of the color [9,10,20,21], Maillard-reaction-induced decrease or increase of antioxidants [9,22,23], decrease of enzyme activity (diastase, invertase), and formation of hydroxymethylfurfural (HMF) [7,10,13,18,19,24]. These attributes also change during long-term normal storage, but heating, especially above 50 °C, highly accelerates these processes. One of the most important indicators of heat treatment is the HMF content, which cannot be higher than 40 mg/kg in honeys (except honeys from tropical regions, where the limit is 80 mg/kg) [2,3,25]. Nevertheless, even at temperatures lower than 60 °C, significant changes can occur in terms of the composition and sensory properties of honey. However, based on the literature, the detection of heat treatment below 60 °C can be challenging when based on HMF content as the sole indicator [16,18,26,27,28].

Currently, multivariate, correlative techniques can offer fingerprint-like information on the analytes, thus representing powerful alternatives to conventional methods. As one of these techniques, near infrared spectroscopy (NIRS) has already been applied to detect the changes deriving from heat treatment [18,29,30]. A new application field of NIRS, called aquaphotomics, aims to check the changes in the water structure of the samples as a result of different perturbations [31]. As honey is a supersaturated solution of sugars in water, the aquaphotomics approach could be a perfect choice for the detection of overheating or other mishandling of honey. Previously, aquaphotomics has been applied to detect changes in honey resulting from adulteration with sugar syrups [32,33,34,35]. However, to the best of the authors knowledge, the aquaphotomics approach has never been applied to evaluate the effect of heat treatment on the compositional changes of honey.

Therefore, this study intends to investigate the effect of heat treatment on the spectral pattern of unifloral honeys using aquaphotomics.

## 2. Results

### 2.1. HMF Content of the Honeys

The initial HMF contents of the three investigated honey types were different: sunflower honeys had an average HMF content of 18.55 ± 0.28 mg/kg, bastard indigo honeys presented 14.68 ± 1.61 mg/kg, and acacia honeys contained 6.96 ± 0.38 mg/kg (Table 1.). These all fulfill the requirements of the legislation (maximum 40 mg/kg) [2,3]. Based on the two-way ANOVA test, the effect of time interval, temperature, and their interaction was found to significantly affect the HMF content of all of the studied honey samples.

No significant difference was found among time intervals within the 40 °C group in sunflower, bastard indigo, and acacia honeys. In the case of the 60 °C group, no clear trend was observed. However, in the case of the honeys heated to 80 °C and 100 °C, the samples heat treated for 60 min had significantly lower HMF content compared to honeys heat treated for 120, 180, and 240 min. Moreover, an increasing trend can be observed in the 80 °C and 100 °C groups with the increase of the treatment time.

In the case of the honeys heat treated for 60 min, the sunflower and acacia honeys treated at 100 °C had higher HMF contents compared to the lower temperatures when the effect of temperature within time intervals was evaluated. Honey samples heat treated for 120 min showed different results in the case of the three honey types. The HMF values of the sunflower honey (120 min group) showed that all the samples were significantly different from each other and the HMF value increased with the increase of the temperature. Results of the bastard indigo honey showed that only the honeys heat treated at 100 °C showed significantly higher HMF contents compared to the lower temperature groups. Acacia honey showed no significant differences between 40 °C and 60 °C, while the 80 °C group had significantly higher HMF contents than the honeys heated at lower temperature levels; the same applied for the 100 °C treated group. The same trend was found in the case of acacia honey for 180 min and 240 min treated honeys. The groups of the 180 min and 240 min treatment of sunflower honey showed that only the 80 °C and 100 °C treated honeys differed significantly from the other temperature levels. In the case of the bastard indigo honey samples heated for 180 min, only the honeys heated at 100 °C showed a significantly higher HMF compared to the honeys heated at lower temperatures. The sunflower honeys heated for 240 min showed the same trend as the bastard indigo and acacia honeys.

Sunflower and acacia honey samples heat treated at 80 °C for 120 min or longer, and the samples heated at 100 °C (all time intervals) showed significantly higher HMF contents compared to control. In the case of the bastard indigo honey, only the samples heated at 100 °C for 120, 180, 240 min had a significantly higher HMF than that of the control. Moreover, it should be highlighted that the limit (40 mg/kg) was reached only in the case of the 100 °C treatment of the bastard indigo and acacia honeys, whereas for sunflower honey, the temperature level of 80 °C and duration of 240 min induced a higher value than the limit. This shows that longer low-level heat treatments and even higher temperatures are not detected by this conventional method, however, even at these lower temperatures (from 60 °C) irreversible changes occur in honeys. Our results are in line with the work of Romanian researchers who also found that more intense HMF formation occurred at higher temperatures, such as 100 °C [24]. Bogdanov [36] also showed that the HMF limit of 40 mg/kg could be reached after one or two days at 60 °C or after a month at 40 °C. However, in a Hungarian study it has been shown that the sensory properties of honey such as the flavor, color, consistency, and odor changed at lower temperatures [37]; the global taste was already affected at 40 °C and 60 °C, based on the results obtained by the potentiometric electronic tongue. Moreover, the group also found that the color changed significantly at 50 °C and 60 °C [18].

The differences in the formation of HMF and the trends observed could originate from the fact that the initial pH, free acidity, amino acid, and sugar composition (especially fructose ratio) of the three honey types are different, which could have a significant impact on the formation of HMF [38].

The obtained results demonstrate the poor ability of HMF to indicate heat treatments of honey samples and thus the need for additional or alternative methods that can provide a higher sensitivity in the detection of such processes that may be misleading to consumers. As such, NIRS and specifically aquaphotomics may be involved in the quality authentication of honeys.

### 2.2. Models of the Botanical Origin of Honey Types

**Figure 1:**
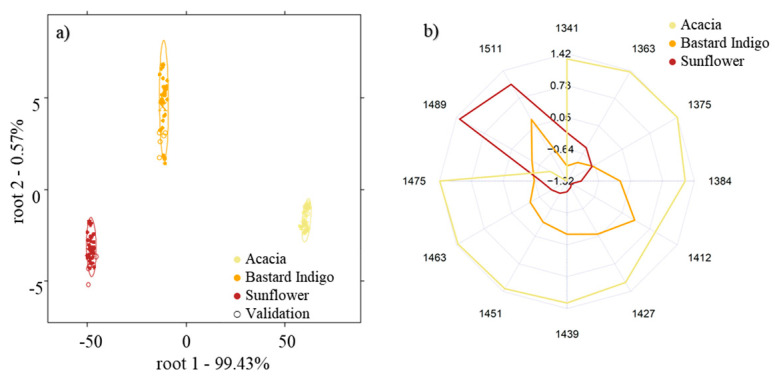
Differentiation of the botanical origin of the three control honeys: (**a**) PCA-LDA score plot for the discrimination of the botanical origin and (**b**) aquagrams of the honey samples of different botanical origins.

The NIRS-based models built for the discrimination of the three types of honey (acacia, sunflower, and bastard indigo) not subjected to any heat treatment (control samples) showed that all the three groups can be separated from each other with 100% accuracy using principal-component-analysis-based linear discriminant analysis (PCA-LDA) (Figure 1a). The aquagram of the honeys also showed completely different spectral patterns for the three honey types. While sunflower honey was mainly characterized by high absorbances at the wavelengths attributed to highly hydrogen-bonded water (1511 nm and 1489 nm), the most abundant water molecular structures for acacia honey consisted of water shells (1363 nm), combinations of antisymmetric and symmetric stretching modes of water and V1- and V2-bonded water [39]. Similar to sunflower honey, bastard indigo honey also presented peaks located at the water matrix coordinates (WAMACs), associated with strongly hydrogen-bounded water molecules, but also at regions attributed to free water (1412 nm), thus attesting the complexity of the physicochemical composition and the crystallization phase of each of the studied honeys (Figure 1b).

### 2.3. Results of the Principal Component Analysis

**Figure 2:**
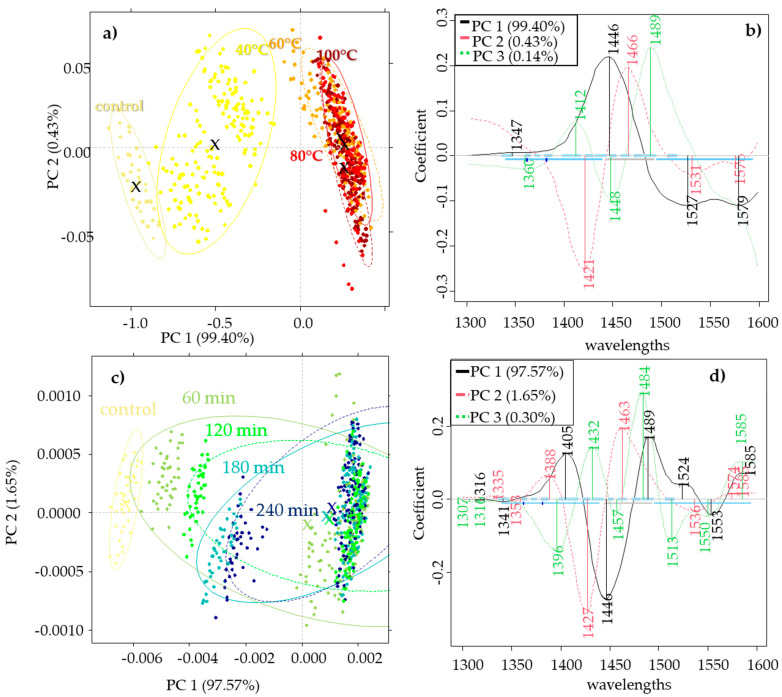
Principal component analysis of the control and heat-treated sunflower honeys: (**a**) PCA score plot for temperature pattern visualization with a Savitzky–Golay smoothing (window size 13) and SNV pretreatment; (**b**) the respective PCA loading plot; (**c**) PCA score plot for the time pattern visualization with a Savitzky–Golay smoothing (window size 13) and Savitzky–Golay 2nd derivative (window size 13, 2nd order derivative) pretreatment; (**d**) the respective PCA loading plot.

Principal component analysis calculated separately for the three honey (acacia, sunflower, bastard indigo) types showed similar results. In the case of the sunflower honey (Figure 2), there was a discrimination pattern through PC1 that described 99.40% of the variance. The group of control samples was completely separated from the scores of honey samples treated at different temperatures (40, 60, 80, 100 °C) and showed a slight overlapping with the honey samples treated at 40 °C. The higher treatment levels overlapped with each other. In this case the 1347 nm, 1446 nm, 1527 nm, and 1576 nm values contributed to the formation of PC1. The PCA model of heating time separation also showed a discrimination tendency through PC1 that described 97.57% of the variance. Values obtained at 1316 nm, 1405 nm, 1446 nm, 1489 nm, 1524 nm, 1553 nm, and 1585 nm contributed to the formation of PC1. In this case, the control slightly overlapped with the honeys treated for 60 min. However, it can also be seen that there are separated subgroups in each time treatment group, which shows the higher effect of the temperature on the spectra of honey.

In the case of the bastard indigo honey, the trends were similar to the sunflower honeys, where the 1534 nm and 1582 nm values contributed to PC1 with the highest weight. The model built to visualize the effect of time intervals showed that the values obtained at 1335 nm, 1448 nm, 1531 nm, and 1579 nm had the highest contribution to PC1, however, in this case, there was no obvious separation trend based on the time intervals.

In the case of acacia honey, the trends were not as obvious as in the case of the other two honey types. The PCA model presenting the temperature pattern showed only a slight separation tendency through the first two principal components. PC1 was mainly obtained by the wavelengths at 1405 nm, 1454 nm, 1524, and 1565 nm. The model depicting the time pattern did not show any clear trend for the separation of the time intervals.

The wavelengths contributing to the model illustrating the temperature level patterns showed that in the case of all the honey types, the regions at 1524–1534 nm and 1582–1585 nm were the most affected ones. The region at 1524–1553 nm can be assigned to the ionic hydrogen bonding vibration in OH−(H_2_O)_2-4_ [40], while the 1582–1585 nm region is characteristic of fructose, sucrose, and glucose [32].

The models showing the pattern of time interval discrimination were also similar where 1316 nm and 1335 nm could be assigned to the weakly H-bonded water. The domination of the free -OH is related to this region. The peaks at 1405 nm, and 1432 nm and 1446 nm are related to the free water and water molecules with one hydrogen bond, respectively, while the region at 1448–1454 nm can be assigned to the OH-(H_2_O)_4,5_ water solvation shell. The assigned wavelengths also revealed that water molecules with four hydrogen bonds were also formed by the heat treatment (1489 nm) [32,41].

Similar results were obtained in a Hungarian study investigating low-level heat treatment of honeys (at 40, 50, and 60 °C for 30, 60, and 120 min) using near-infrared spectroscopy in the range of 950–1630 nm. In this research, the sunflower honeys showed a similar trend based on the PCA, the separation of the control sample was clear and a slight overlapping was found with the 40 °C treated samples, however, the 60 °C treated honeys separated completely from the control honey [18]. However, no clear separation tendency was observed for the acacia honeys.

### 2.4. Results of the PCA-LDA Analysis

The general (including all the sublevels of time and temperature treatments) PCA-LDA models, built separately for each honey type, did not provide strong classification accuracies in the case of the different honey types. The general model built for the classification of the different temperature levels showed average training (recognition) and cross-validation (prediction) accuracies of 80.18% and 68.79%, 82.24% and 75.95%, and 64.82% and 48.44% for the sunflower, bastard indigo, and acacia honeys, respectively. The models of the time interval classifications weren’t effective with 52.70% and 35.29%, 63.78% and 41.33%, 47.38% and 25.93% accuracies for the sunflower, bastard indigo and acacia honeys, respectively. However, the detailed (i.e., built for the classification of time intervals within temperature levels, or built for the classification of temperature levels within time intervals) models provided better classification accuracies.

**Figure 3:**
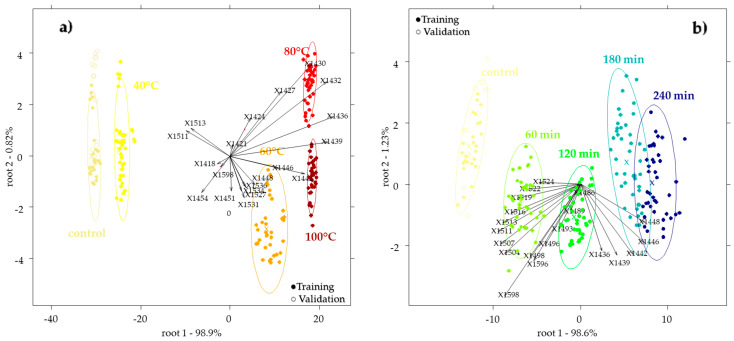
PCA-LDA score plot of the sunflower honey with the 20 most important loadings: (**a**) temperature classification model of honeys treated for 60 min with a Savitzky–Golay smoothing (window size 21) and SNV pretreatment; (**b**) time classification model of honey heated at 40 °C with Savitzky–Golay smoothing (window size 13), MSC and detrending pretreatment.

In Figure 3a,b, an example of the PCA-LDA score plot for the discrimination of the temperature or time interval of the applied heat treatment within the time or temperature groups, respectively, is showcased (detailed models). Loadings on the plot show the 20 wavelengths that contributed the most to the separation of the groups.

In each of the models, the presented results were chosen from 41 pretreatment combinations after a leave-one-sample-out cross-validation, based on the best validation accuracy.

Detailed temperature-level PCA-LDA models (Table 2 upper part) provided the best results in the case of the sunflower honey. Honeys heated for 240 min showed a 100% classification accuracy of the temperature levels. The models of the honeys heated for 60 min showed a slightly lower accuracy (97.47%) after validation. The temperature groups 60 °C and 80 °C also provided worse results; however, in the case of all the models, the control was classified correctly. The classification models of bastard indigo weren´t accurate, but similarly, the honeys heated for four hours showed the best classification of temperature. In this case, all the models provided 100% correct classification of the control. Models of the acacia honey weren´t as performant as those of the other two groups, where the control classification accuracy was higher in the honeys heated for longer time intervals.

The detailed models for the classification of the time levels within temperature groups (Table 2 lower part) provided the best training and validation accuracies in the case of the sunflower honey, followed by bastard indigo, and acacia samples. In general, these models were worse than the models of the temperature classification within time intervals. The validation accuracies of the sunflower and bastard indigo honeys showed similar trends, where the best models were obtained for the time interval classification within 40 °C, followed by the model at 100 °C, while the models of the other two temperatures were weaker. In the case of acacia honey, the best model was achieved for the time interval classification within 80 °C, while similarly to bastard indigo, the worst model was obtained for the 60 °C treated group. The control was classified correctly in all the models of sunflower and bastard indigo. The models of the acacia honey provided the best results for the control: a correct classification for the models with 80 °C, followed by 100 °C, 60 °C, and 40 °C.

Poor results were also obtained for acacia samples in a Hungarian study [18]. This could be due to the lower nutritional content and the different crystallization phases of the honey samples. These results support the findings of Segato et al., 2019, who also found that the changes in NIR spectra as a result of heat treatment are highly phase-related, which could come from the fact that the scattering of the crystals is different in the different crystallization phases [42].

### 2.5. Results of the Partial Least Square Regression of Honeys

The results presented here were chosen from 41 pretreatment combinations after leave-one-sample-out cross-validation, based on the best R^2^CV.

Results of the general (including all the temperature levels or time intervals) PLSR models of the three honey types provided the best results in the case of the sunflower honeys, followed by bastard indigo, and acacia honeys. The prediction of temperature provided a R^2^CV of 0.81 and RMSECV of 10.70 °C, R^2^CV of 0.76 and RMSECV of 11.82 °C, R^2^CV of 0.36 and RMSECV of 19.00 °C for the sunflower, bastard indigo, and acacia honeys, respectively.

The prediction model of time intervals was much worse where in all the honey types, the R^2^CV was lower than 0.26 and the error RMSECV was higher than 60 min. Therefore, more detailed models were calculated, their corresponding parameters are shown in Supplementary Table S1.

The detailed temperature level PLSR models built for the prediction of the applied temperature within the four studied time intervals (Supplementary Table S1 upper part) provided the best results in the case of sunflower honey. The residual prediction deviation (RPD) values were between 2.35 and 3.96 while the R^2^CV was higher than 0.80 in all four models. The best prediction accuracy was obtained for the 240 min group. The model parameters of the bastard indigo honey were worse than in the case of the sunflower honey, however, the RPDCV values were >2.0 in all cases and the R^2^CV values were between 0.76 and 0.88. Similarly, the 240 min group provided the best results. The prediction models of the acacia honey were worse than the results of the two other honey types. The RPD values were below 2.0 and the R^2^CV values ranged between 0.29 and0.68. The best models were obtained in the case of the 60 min group, while those built for the 180 min group were less effective.

The detailed time interval PLSR models (Supplementary Table S1 lower part) also provided better results in the case of the sunflower honey compared to the bastard indigo and acacia honeys. The best results were obtained for the 40 °C group with an R^2^CV value of 0.97 and RPDCV > 5.0. Lower prediction accuracies were obtained for the 80 °C group with an R^2^CV of 0.83 and RPDCV of 2.45. In the case of the bastard indigo honey, the model of the 40 °C group provided the best results (R2CV = 4.13 and RPDCV = 4.13). The 60 °C and 80 °C groups models weren´t as accurate with R2CV < 0.5. For acacia honey, the most accurate prediction was achieved in the case of the 100 °C group with R2CV and RPDCV values of 0.83 and 2.45, respectively.

Summarizing, similar to the results of PCA-LDA, the best results were achieved in the case of the sunflower followed by the bastard indigo and acacia honeys.

### 2.6. Results of the Aquagrams Visualizing the Effect of Temperature and Time Intervals

**Figure 4:**
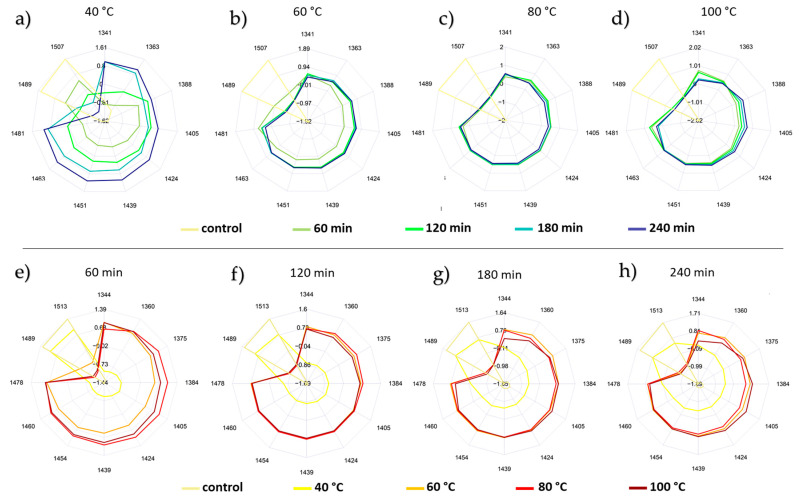
Aquagrams of the sunflower honey: (**a**–**d**) aquagrams showing pattern of time intervals; (**e**–**h**) aquagrams showing the pattern of temperature levels.

**Figure 5:**
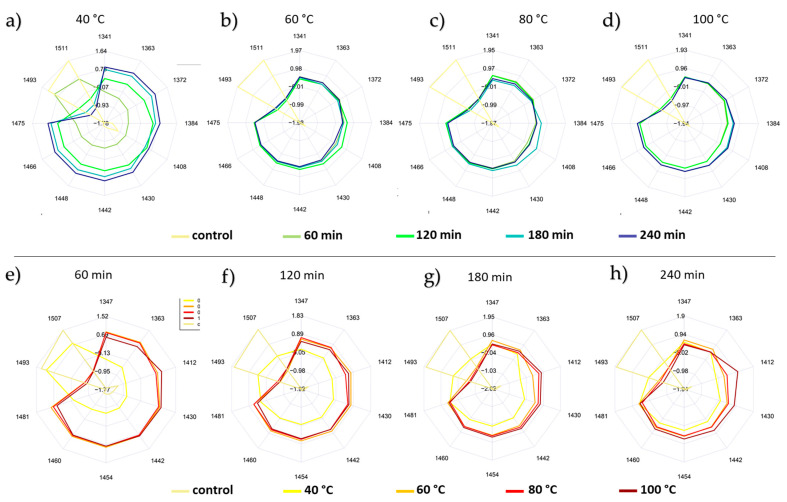
Aquagrams of the bastard indigo honey: (**a**–**d**) aquagrams showing pattern of time intervals; (**e**–**h**) aquagrams showing the pattern of temperature levels.

**Figure 6:**
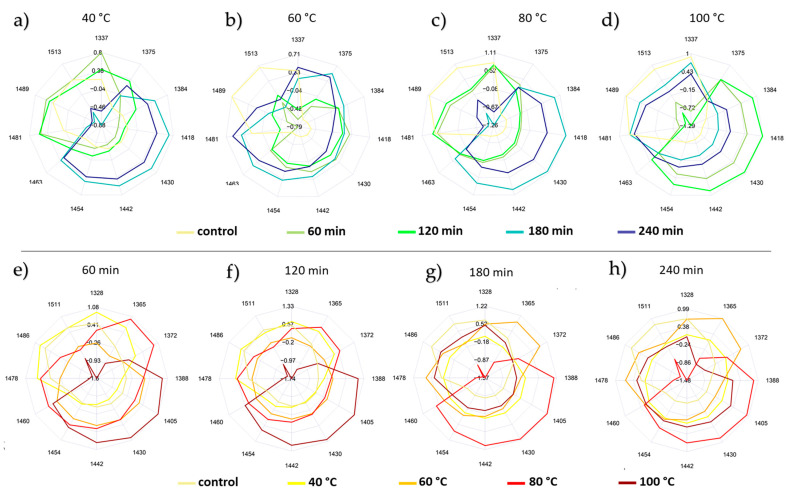
Aquagrams of the acacia honey: (**a**–**d**) aquagrams showing pattern of time intervals; (**e**–**h**) aquagrams showing the pattern of temperature level.

The aquagrams plotted at the water matrix coordinates for each honey type as predefined based on the subtraction spectra, the PCA loadings of the temperature (Figure 2a) and time visualization models (Figure 2b), as well as the most contributing wavelengths of the previously presented PCA-LDA models (Figure 3a,b) and PLSR regression models (Supplementary Figure S1b,d), are presented in Figure 4 (sunflower honey), Figure 5 (bastard indigo honey), and Figure 6 (acacia honey).

To better understand the effect of the heat treatment parameters (time and temperature) on the water spectral pattern of each honey type, their respective aquagrams were inspected while fixating one parameter and changing the other.

As portrayed in Figure 4, across the different temperature levels (Figure 4 upper part) and time intervals (Figure 4 lower part), the control group for sunflower honey is markedly distinguished from the heat-treated samples.

Within each time interval, and as the applied temperature increases, lower absorbances at the highly hydrogen-bonded bands (1489 nm to 1513 nm) and higher ones at the free water conformations are observed. This pattern is particularly pronounced at the 60 min time frame (Figure 4e), where exceeding the temperature of 40 °C (maximum allowed) to reach 60 °C translates into a larger scale change of the spectral pattern.

As can be seen from the aquagrams, at the lowest applied temperature of 40 °C, the heating period can have a significant influence on the water structure (Figure 4a). This influence can still be noticed at 60 °C (Figure 4b), but this change towards the less hydrogen-bounded water structure is not that prominent anymore when higher temperatures are considered (Figure 4c,d).

Following the same rationale when analyzing the bastard indigo honey samples (Figure 5), the impact of the temperature (Figure 5 upper part) and time interval (Figure 5 lower part) of the heat treatment is reflected in the aquagram through the liquefaction of the initially bonded water into more free water structures (Figure 5). Again, the time interval can be considerably impactful when it comes to affecting the existing water structures at 40 °C (Figure 5a), but not as much when the honey was heated up to 60 °C, 80 °C, and 100 °C (Figure 5b–d).

Explanation of the heat treatment effect on acacia honey (Figure 6), on the other hand, was less manageable. While the alteration of the water conformation is clearly reflected in the corresponding aquagram (Figure 6), these changes do not follow a logical pattern either along the varying time intervals (Figure 6 upper part), nor on the basis of the applied temperatures levels (Figure 6 lower part).

Such a behavior can be justified by the inherent differences in terms of the crystallization state and the physicochemical features of acacia honey compared to sunflower and bastard indigo honey samples [43]. This peculiar state has already been proven when the water spectral patterns of the three studied honey types were investigated in the absence of heat treatment (control group), where acacia, unlike the other two honey types, had abundant free water and less hydrogen-bonded water molecules. Segato et al. [42] also found that the changes of the spectral pattern in honeys are phase-related, and he also found that honey that was initially in liquid state (such as acacia in this study) showed less changes compared to the completely crystallized (like sunflower in our case) and less crystallized samples (like bastard indigo honey in our case). The HMF content of the honeys also showed similar trends: the HMF formation was the most intense in the sunflower honey, followed by bastard indigo and acacia honeys (Table 1). However, it should be noted that the NIRS seemed to be more sensitive to the changes from the effect of heat treatment, as the aquagrams, PCA-LDA, and PCA models showed the changes even at lower levels, while in the case of the HMF, only honeys heated at 80 °C for at least 120 min and 100 °C showed significant changes compared to the control honeys.

These findings do illustrate that the potential adulteration of honey by means of heat processing can be reflected in the respective aquagrams. Not only can this approach indicate—to some extent—the differences induced by the temperature levels at different time periods but it can also confirm the complexity of detecting such instances that are not only dictated by the thermal treatment parameters but can highly depend on the initial crystallization state of the honey.

## 3. Materials and Methods

### 3.1. Honey Samples

Three types of unifloral honey were collected from reliable beekeepers. The honeys were not processed or heat treated after the collection from the beehive. Sunflower (*Helianthus annuus)*, bastard indigo (*Amorpha fruticosa*), and acacia (*Robinia pesudoacacia*) honeys were used in the study. Three bottles per honey type, each of 1 kg, were collected from the three honeys (nine bottles in total). The three individual bottles were used as replicates (R) in the experiments.

The samples were portioned out to 100 mL glass sample holders with a closing polyethylene cap; 50 g of honey was weighed into the glasses. After portioning, the samples were processed by heat treatment. The samples were heated to 40 °C, 60 °C, 80 °C, or 100 °C and kept at temperature for 60, 120, 180, or 240 min each. This resulted in 17 heat treatment levels (including the untreated control), where 3 samples were created for each treatment level (the three replicates originated from the three bottles), resulting in 51 samples per type and 153 samples in total. The heat treatment was performed in a drying chamber.

### 3.2. Hydroxymethylfurfural Measurements of Honey

HMF determination of honey was performed according to the guideline of the International Honey Commission [44], using the spectrophotometric Winkler method. A Thermo Helios Alpha (Thermo Fischer Scientific Inc., Waltham, MA, USA) UV–VIS spectrophotometer (±0.001 units of absorbance, 1 cm light path) at 550 nm, using quartz cuvettes, was applied for the HMF quantifications.

### 3.3. Near-Infrared Spectroscopy Measurements

The NIRS measurements were performed using a handheld NIR-S-G1 Spectrometer (InnoSpectra Co., Hsinchu, Taiwan). Spectra were recorded in the range of 900–1700 nm, with 3 nm wavelength step in a transflectance setup. The layer thickness of the sample in the cuvette was 0.4 mm, which ensured that the maximum absorbance values did not exceed two absorbance unit. Each sample was measured three times applying three different fills with five consecutive scans, each resulting in 15 spectra per sample, and a total of 45 spectra per heat treatment level (per unifloral honey). Samples were measured in randomized order. After each five samples, a reference water sample was measured.

### 3.4. Statistical Analyis

#### 3.4.1. ANOVA Analysis of the HMF Content

The HMF results of the honey samples were analyzed using ANOVA. Before the building of the ANOVA models, assumptions such as the normality (using Shapiro–Wilk test) and homogeneity of the variances (using Levene’s test) were checked. To calculate which heat treatment levels differed significantly from the control, a one-way ANOVA test was performed. Additionally, a two-way ANOVA was used to check if the temperature level, time interval, and their interaction (time interval*temperature level) have a significant effect on the HMF formation in honeys. In case of a significant interaction, the significant differences within temperature and within time intervals were analyzed at the *p* < 0.05 significance level. Moreover, in the case of a significant ANOVA model, a post hoc test was applied the following way: when the homogeneity of the variances was assumed, Tukey’s test was used and if it was not assumed, then Games–Howell’s test was used for the pairwise comparison [45].

#### 3.4.2. Spectral Range and Spectral Pretreatments

The spectral range of 1300–1600 nm was applied throughout the analysis. The evaluations were performed according to the protocol of aquaphotomics described by Tsenkova et al., 2018 [39]. The three honey types were evaluated separately. Prior to the data analysis, an outlier detection was applied on the dataset using the built-in outlier detection function of the aquap2 package [46].

After the inspection of the raw spectra, spectral pretreatment optimization was performed using 41 pretreatment combinations. A Savitzky–Golay (Sgol) smoothing (2nd order polynomial) with different window sizes (21, 17, or 13) and derivation varieties (no derivation, 1st, and 2nd derivative) was applied to reduce the noise in the spectra and to reveal overlapping peaks. Standard normal variate or multiplicative scatter correction was applied to reduce the baseline shift. Detrending and the aforementioned techniques were tested in single, double, or triple variations.

#### 3.4.3. Modellings of the NIR Dataset

##### Principal Component Analysis—PCA

Principal component analysis was performed on the three honey types separately to reveal the patterns of time interval and temperature level. The PCA plots were colored according to the temperature level and the time interval. The models of the PCA were built using the best pretreatment obtained during the PCA-LDA of the general models (including all the temperature or time intervals) built for the temperature and time discrimination. This resulted in two models per honey type—one model for the presentation of the temperature pattern, and one for the time interval pattern.

##### PCA-Linear Discriminant Analysis—PCA-LDA

The hybrid PCA-LDA analysis was used to build the classification models. All the models were built using the 41 pretreatment combinations. As a first step, a classification model was built for the discrimination of the botanical types, then general models were built for the classification of the temperature groups (general temperature model) and for the classification of time groups (general time interval model), including all the sublevels of time or temperature treatments. However, due to the high interaction of temperature and time interval, models were also built for the classification of time intervals within each of the temperature groups (detailed time interval models) and for the temperature levels within each time interval (detailed temperature models). It resulted in a total of eight models per honey type (four models for the time interval discrimination within the temperature groups and four models for the temperature-level discrimination within the time intervals), and 24 models in total after choosing the models with the highest validation accuracy. A PC number optimization was also performed where the models with the highest validation accuracy and at the same time lowest difference between training and validation accuracy were chosen. The final models were validated using a leave-one-sample-out validation (LOSO), where all scans of one replicate of the samples were left out in each iteration step of the cross-validation.

##### Partial Least Square Regression (PLSR)

A partial least square regression was first performed on the three honey types separately for the prediction of time intervals and temperatures. A PLSR was also performed to regress on the time interval within each temperature level (detailed time interval models) and temperature level within each time interval (detailed temperature-level models). This also resulted in 8 models per honey type, and a total of 24 models, similarly to the PCA-LDA analysis. The correlation between the actual and predicted values was reported by the R2C and R2CV values. The error of the prediction was calculated using the root mean square error of the training (RMSEC) and validation (RMSECV). Besides the RMSE values, the residual prediction deviation (RPD) was also calculated for the training (RPDC) and validation (RPDCV). All the models were validated with a leave-one-sample-out (LOSO) cross-validation, where the spectra of one replicate of the samples were left out. In the case of the PLRS models, the pretreatment optimization was also performed, where the models with the best model parameters (based on the RMSECV, R2CV, and RPDCV) were chosen.

##### Subtraction Spectra

Subtraction spectra were calculated for the time interval subsets (detailed time interval models) and temperature level subsets (detailed temperature level models), where the control sample was subtracted from the others to reveal the wavelengths showing the greatest changes occurring upon the heat treatment temperature or time, respectively. The subtraction was performed on spectra that were pretreated with MSC and Savitzky–Golay smoothing (2nd order polynomial, window size of 21) with a 2nd derivation separately.

#### 3.4.4. Determination of the Water Matrix Coordinates for the Aquagrams

The wavelengths belonging to the water matrix coordinates were chosen from the PCA loadings, PCA-LDA weights, PLRS regression vectors, and subtraction spectra. The wavelengths presenting the highest change due to the temperature or the time interval perturbation were collected. The assigned wavelengths were collected and grouped to the 12 water matrix coordinates (WAMACs) defined by Tsenkova [47] and the wavelengths presented the most frequently (within one WAMAC) were chosen for the calculation of the classic aquagrams.

Calculation of the aquagrams was performed in such a way as to illustrate the spectral pattern changes as a result of time intervals within one temperature level (detailed time interval models) and also to illustrate the effect of temperature level within one time interval (detailed temperature models). In total, 24 aquagrams were calculated, eight for each of the three honey types.

## 4. Conclusions

In this study, three different honey types were analyzed in terms of the effect of the heat treatment at different temperatures and different time intervals. The impact of the applied processing was measured by determining the HMF content and by NIRS. The temperatures and their interaction highly influenced the HMF formation of honey; however, the temperature level had a higher effect than the time interval. The dynamics of HMF formation differed for the three honey types, which underlines the importance of the initial physicochemical composition, defined by the origin of the honey, in the HMF production. However, only honeys heated at 80 °C and 100 °C provided significant changes in HMF content, which shows the limited ability of HMF as a sole indicator of honey heat treatment.

The PCA-LDA models provided better results for the temperature-level classifications than for the time interval. In general, sunflower honey provided better results on the classification and prediction accuracies than the other two honey types. In all cases, the worst model parameters were obtained for the acacia honeys. It can be concluded that the NIRS was more sensitive than HMF in detecting the effect of heat treatment. Nevertheless, these results indicate that the changes generated byheat treatment are related to the initial crystallization phase (as the sunflower was completely crystallized, bastard indigo was mid-crystallized and the acacia was in liquid form) and the composition of the samples; therefore, it is not possible to set up general principles for the detection of heat processing in honeys. On the other hand, our results prove that even at a low temperature treatment (40 °C), measurable changes occur in the spectra of the honey, and that these spectral changes are mainly related to the transformation of the highly bonded water to less H-bonded water or free water. Besides the water structure, the sugar composition was also modified, which could be concluded from the changes in the wavelength range of 1580–1590 nm.

Our research reveals the potential of NIRS and specifically aquaphotomics in the detection of the changes induced in the water structure of honey by heat treatment. In the future, it would also be important to see how the spectral pattern is affected as a result of heat treatment after recrystallization and storage.

## Figures and Tables

**Table 1:** Results of the HMF content of the honey samples from the heat treatment experiment.

Hydroxymethylfurfural Content, mg/kg						
		Control	40 °C	60 °C	80 °C	100 °C
**Sunflower**	**Control**	18.5 ± 0.3				
	**60 min**		20.2 ± 1.5 ^aA^	16.2 ± 1 ^aA^	17.6 ± 0.2 ^aA^	40.3 ± 0.8 ^aB^*
	**120 min**		17.3 ± 1.3 ^aA^	20.5 ± 0.7 ^bB^	31.8 ± 1.3 ^bC^*	155.1 ± 2.7 ^bD^*
	**180 min**		18.4 ± 1.6 ^aA^	19.9 ± 1.8 ^bA^	37.2 ± 0.6 ^cB^*	241.5 ± 7.4 ^cC^*
	**240 min**		17.5 ± 1.4 ^aA^	19.5 ± 2 ^abA^	52 ± 2.7 ^dB^*	463.6 ± 28.3 ^dC^*
Bastard indigo **	**Control**	14.7 ± 1.6				
	**60 min**		14.1 ± 2.8 ^aAB^	18 ± 2.3 ^abB^	11.9 ± 1.1 ^aA^	16.7 ± 0.9 ^aAB^
	**120 min**		15.1 ± 3.5 ^aA^	15.8 ± 0.6 ^abA^	14.3 ± 1 ^bA^	81.4 ± 4 ^bB^*
	**180 min**		15.7 ± 1.1 ^aA^	21.1 ± 3.5 ^bA^	19.8 ± 0.6 ^cA^	146.4 ± 2.3 ^cB^*
	**240 min**		12.9 ± 1.4 ^aA^	13.7 ± 1.3 ^aA^	28.2 ± 1.1 ^dB^	306 ± 17.8 ^dC^*
**Acacia**	**Control**	7.0 ± 0.4				
	**60 min**		9.1 ± 1.3 ^aA^	7.7 ± 0.3 ^aA^	8 ± 0.4 ^aA^	16.1 ± 1.7 ^aB^*
	**120 min**		8 ± 0.6 ^aA^	8.8 ± 1.4 ^aA^	13.3 ± 0.9 ^bB^*	44.7 ± 4.3 ^bC^*
	**180 min**		8.6 ± 1 ^aA^	9.6 ± 0.3 ^aA^	12.2 ± 0.8 ^bB^*	89.1 ± 2.8 ^cC^*
	**240 min**		10 ± 1.1 ^aA^	9.6 ± 0.9 ^aA^	18.8 ± 2.4 ^cB^*	211.6 ± 5 ^dC^*

Letters represent the significant differences between the samples based on the results of an ANOVA test and pairwise comparisons at *p* < 0.05: lowercase letters (a,b,c,d) stand for the differences between time intervals (columns) within a temperature level; capital letters (A,B,C,D) are for the differences between temperature levels within time intervals (rows); * are for a significantly different level compared to the control sample. ** Results of bastard indigo were previously presented at a conference [29].

**Table 2:** PCA-LDA classification accuracies of the detailed temperature level and time interval classification models.

Temperature Classification within Time Group					
Honey	Subgroup	Pretreatment	Training %	Validation %	Control %
**Sunflower**	within 60 min	sgol@2-21-0_snv	99.93	97.47	100
	within 120 min	sgol@2-13-0_sgol@2-13-2	97.74	87.55	100
	within 180 min	sgol@2-17-0	99.82	91.37	100
	within 240 min	sgol@2-13-0_sgol@2-17-2	100	100	100
**Bastard indigo**	within 60 min	sgol@2-17-0_deTr	81.11	62.38	100
	within 120 min	sgol@2-17-0_deTr	88.99	78.56	100
	within 180 min	sgol@2-17-0_deTr	92.74	83.71	100
	within 240 min	sgol@2-13-0_sgol@2-21-1	97.92	93.22	100
**Acacia**	within 60 min	sgol@2-21-0_deTr_msc	85.74	66.30	51.28
	within 120 min	sgol@2-13-0_sgol@2-17-1	91.11	59.94	69.23
	within 180 min	sgol@2-13-0_msc	91.56	72.17	76.92
	within 240 min	sgol@2-13-0_sgol@2-17-1	84.69	66.10	76.92
**Time Classification within Temperature Group**					
**Honey**	**Subgroup**	**Pretreatment**	**Training %**	**Validation %**	**Control %**
**Sunflower**	within 40 °C	sgol@2-13-0_deTr_msc	98.63	96.36	100
	within 60 °C	sgol@2-17-0_sgol@2-21-1	90.58	72.47	100
	within 80 °C	sgol@2-21-0_sgol@2-13-1	84.80	57.27	100
	within 100 °C	sgol@2-13-0_sgol@2-13-2	89.95	72.21	100
**Bastard indigo**	within 40 °C	sgol@2-17-0_deTr	97.05	95.16	100
	within 60 °C	sgol@2-17-0	76.15	47.94	100
	within 80 °C	sgol@2-13-0_sgol@2-13-2	82.80	55.40	100
	within 100 °C	sgol@2-17-0	89.52	61.66	100
**Acacia**	within 40 °C	sgol@2-21-0_sgol@2-13-1	71.19	42.99	56.41
	within 60 °C	sgol@2-21-0_deTr_msc	62.21	31.96	71.79
	within 80 °C	sgol@2-17-0_msc	82.77	58.66	82.05
	within 100 °C	sgol@2-13-0_sgol@2-17-2	94.79	72.64	79.49

Each row represents the best model chosen from the 41 pretreatment combinations, the average classification accuracies for the training, validation, and the control correct classification are computed from the leave-one-sample-out cross validation confusion tables. sgol@x-y-z means Savitzky–Golay smoothing, where x denotes the polynomial order, y the window size and z the order of derivation; msc denotes multiplicative scatter correction; snv denotes standard normal variate; detr denotes detrending.

## Data Availability

The data presented in this study are available on request from the corresponding author. The data are not publicly available, due to privacy reasons.

## Supplementary Materials

The following are available online, Table S1: Results of the partial least square regression models built for the prediction of time interval within temperature group and temperature within time intervals. Figure S1: Partial least square regression plot of the sunflower honeys: (a) regression plot of honeys heated for 60 min regressed on the temperature levels with Savitzky–Golay smoothing (window size: 17) and detrending; (b) the respective regression vector; (c) regression plot of honeys heated at 40 °C regressed on the time intervals with Savitzky–Golay smoothing (window size: 17), detrending, and MSC; (d) the respective regression vector.

## Sample Availability

Not available.
