# Supplementary material for: Revealing the Effect of Heat Treatment on the Spectral Pattern of Unifloral Honeys Using Aquaphotomics
Source: Molecules. 2022 Jan 25;27(3):780. doi: 10.3390/molecules27030780 (PMC8839790; doi:10.3390/molecules27030780)
Supplement: Supplementary file 1 [file molecules-27-00780-s001.zip › molecules-1527588-supplementary materials.pdf]

Table S1. Results of the partial least square regression models built for the prediction of time interval within temperature group and temperature within time intervals

	Subgroup	Pretreatment	NrLV	NrObs	R^2Tr	RMSEC	RPDC	R^2CV	RMSECV	RPDCV
**Detailed temperature level prediction models**										
**Sunflower**	within 60 min	sgol@2-17-0_deTr	5	160	0.93	7.09	3.89	0.89	9.25	2.98
	within 120 min	sgol@2-21-0_sgol@2-17-2	5	157	0.87	9.85	2.80	0.82	11.76	2.35
	within 180 min	sgol@2-17-0_deTr_snv	5	160	0.94	6.72	4.08	0.90	8.59	3.19
	within 240 min	sgol@2-21-0_deTr_msc	5	149	0.96	5.63	4.76	0.94	6.78	3.96
**Bastard indigo**	within 60 min	sgol@2-21-0_snv	5	165	0.84	10.60	2.54	0.77	13.00	2.07
	within 120 min	sgol@2-13-0_msc	5	159	0.86	9.97	2.66	0.77	12.58	2.11
	within 180 min	snv	5	169	0.89	8.84	3.05	0.76	13.03	2.07
	within 240 min	sgol@2-13-0_sgol@2-13-2	5	139	0.92	7.51	3.60	0.88	9.35	2.89
**Acacia**	within 60 min	sgol@2-17-0_msc	5	156	0.79	12.44	2.18	0.68	15.36	1.77
	within 120 min	sgol@2-21-0	5	154	0.73	13.65	1.94	0.65	15.65	1.69
	within 180 min	sgol@2-17-0_deTr_msc	5	149	0.51	19.20	1.43	0.29	23.13	1.19
	within 240 min	sgol@2-13-0_sgol@2-13-1	5	162	0.73	13.52	1.94	0.60	16.52	1.59
**Detailed time-period prediction models**										
	**Subgroup**	**Pretreatment**	**NrLV**	**NrObs**	**R^2Tr**	**RMSEC**	**RPDC**	**R^2CV**	**RMSECV**	**RPDCV**
**Sunflower**	within 40 °C	sgol@2-17-0_deTr_msc	5	171	0.98	11.62	7.32	0.97	14.49	5.87
	within 60 °C	sgol@2-21-0_sgol@2-17-2	5	151	0.91	26.31	3.28	0.83	35.17	2.46
	within 80 °C	sgol@2-13-0_deTr	5	155	0.88	28.83	2.93	0.83	34.42	2.45
	within 100 °C	sgol@2-17-0_sgol@2-21-1	5	165	0.91	24.56	3.41	0.89	27.84	3.00
**Bastard indigo**	within 40 °C	sgol@2-13-0_deTr_snv	4	156	0.96	17.24	4.94	0.94	20.60	4.13
	within 60 °C	sgol@2-13-0	1	181	0.55	58.12	1.49	0.46	63.75	1.36
	within 80 °C	sgol@2-13-0	3	167	0.54	56.96	1.47	0.42	63.60	1.32
	within 100 °C	sgol@2-17-0_snv	5	148	0.92	24.62	3.46	0.87	30.27	2.81
**Acacia**	within 40 °C	sgol@2-21-0_sgol@2-21-1	5	162	0.61	53.44	1.61	0.50	60.59	1.42
	within 60 °C	sgol@2-21-0_sgol@2-17-2	4	160	0.61	53.96	1.60	0.47	62.59	1.38
	within 80 °C	sgol@2-21-0	5	158	0.79	39.47	2.16	0.67	49.02	1.74
	within 100 °C	sgol@2-21-0_sgol@2-13-1	5	145	0.87	30.71	2.76	0.83	34.62	2.45

Each row represents the best model chosen from the 41 pretreatment combinations, model parameters are computed using leave-one-sample-out cross-validation

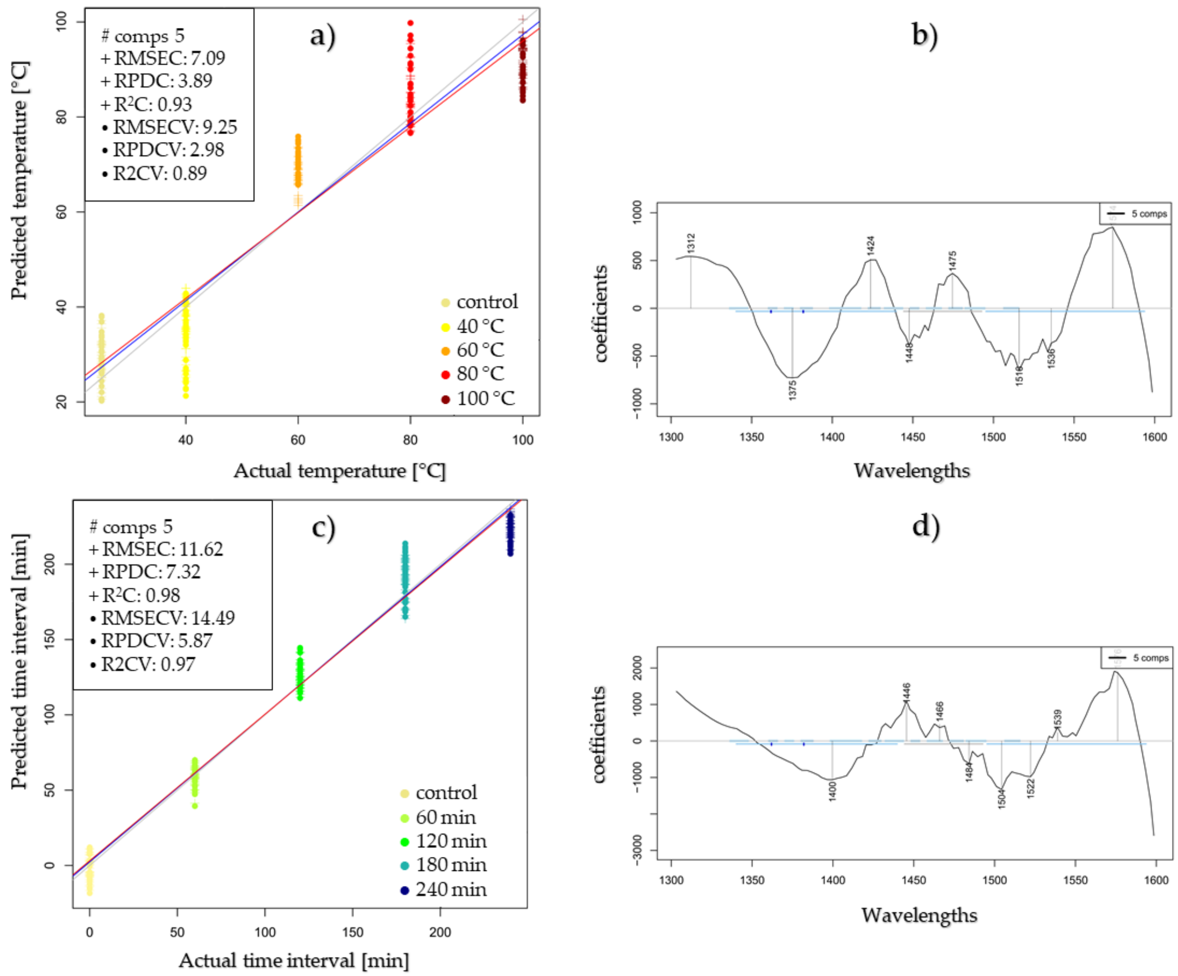

Figure S1: Partial least square regression plot of the sunflower honeys: **a**) regression plot of honeys heated for 60 min regressed on the temperature levels with Savitzky–Golay smoothing (window size: 17) and detrending; **b**) the respective regression vector; **c**) regression plot of honeys heated at 40 °C regressed on the time intervals with Savitzky–Golay smoothing (window size: 17), detrending, and MSC; **d**) the respective regression vector.
